# 3-Phenyl­sulfanyl-4-phenyl­sulfonyl-1,2,5-oxadiazole 2-oxide

**DOI:** 10.1107/S1600536810043060

**Published:** 2010-11-10

**Authors:** Giuliana Gervasio, Domenica Marabello, Federica Bertolotti

**Affiliations:** a Dipartimento di Chimica I, F.M. e Centro CrisDi, University of Turin, Via P. Giuria 7, 10125, Torino, Italy

## Abstract

In the title compound, C_14_H_10_N_2_O_4_S_2_,the furoxan heterocyclic ring and the two S atoms are almost co-planar, with a mean deviation of 0.036 Å. The bond lengths in the penta­gonal  ring show electron delocalization and the furoxan N—O bond length is quite short [1.211 (3) Å]. The dihedral angles between the central ring and pendant phenyl rings are 78.05 (14) and 84.28 (2)°.

## Related literature

This is part of a study on phenyl­sulfonyl-substituted furoxans as inter­mediates for the synthesis of new functionalized furoxans with potential biological properties as *N*,*O*-donors. For details of the synthesis, see: Sorba *et al.* (1996 ▶); Tosco *et al.* (2004 ▶). For a related structure, see: Dutov *et al.*(2007 ▶).
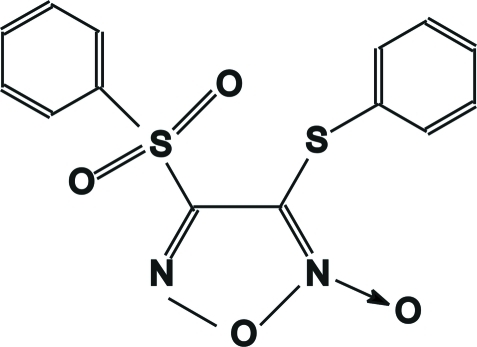


## Experimental

### 

#### Crystal data

C_14_H_10_N_2_O_4_S_2_
*M* *_r_* = 334.36
Orthorhombic, 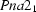

*a* = 15.0182 (2) Å
*b* = 5.5402 (1) Å
*c* = 17.8280 (2) Å
*V* = 1483.36 (4) Å^3^
*Z* = 4
Cu *K*α radiation
μ = 3.44 mm^−1^
*T* = 293 K
0.20 × 0.16 × 0.14 mm

#### Data collection

Gemini R Ultra diffractometer
Absorption correction: multi-scan (*CrysAlis RED*; Oxford Diffraction, 2008 ▶) *T*_min_ = 0.836, *T*_max_ = 1.000
7933 measured reflections
2255 independent reflections
2134 reflections with *I* > 2σ(*I*)
*R*_int_ = 0.023
θ_max_ = 62.2°

#### Refinement

*R*[*F* ^2^ > 2σ(*F* ^2^)] = 0.030
*wR*(*F* ^2^) = 0.084
*S* = 1.05
2255 reflections
199 parameters
1 restraint
H-atom parameters constrained
Δρ_max_ = 0.20 e Å^−3^
Δρ_min_ = −0.13 e Å^−3^
Absolute structure: Flack (1983 ▶), 1039 Friedel pairs
Flack parameter: 0.010 (17)

### 

Data collection: *CrysAlis CCD* (Oxford Diffraction, 2008 ▶); cell refinement: *CrysAlis RED* (Oxford Diffraction, 2008 ▶); data reduction: *CrysAlis RED*; program(s) used to solve structure: *SHELXS97* (Sheldrick, 2008 ▶); program(s) used to refine structure: *SHELXL97* (Sheldrick, 2008 ▶); molecular graphics: *XP* in *SHELXTL* (Sheldrick, 2008 ▶); software used to prepare material for publication: *SHELXL97*.

## Supplementary Material

Crystal structure: contains datablocks I, global. DOI: 10.1107/S1600536810043060/cv2780sup1.cif

Structure factors: contains datablocks I. DOI: 10.1107/S1600536810043060/cv2780Isup2.hkl

Additional supplementary materials:  crystallographic information; 3D view; checkCIF report

## Figures and Tables

**Fig. 1.**
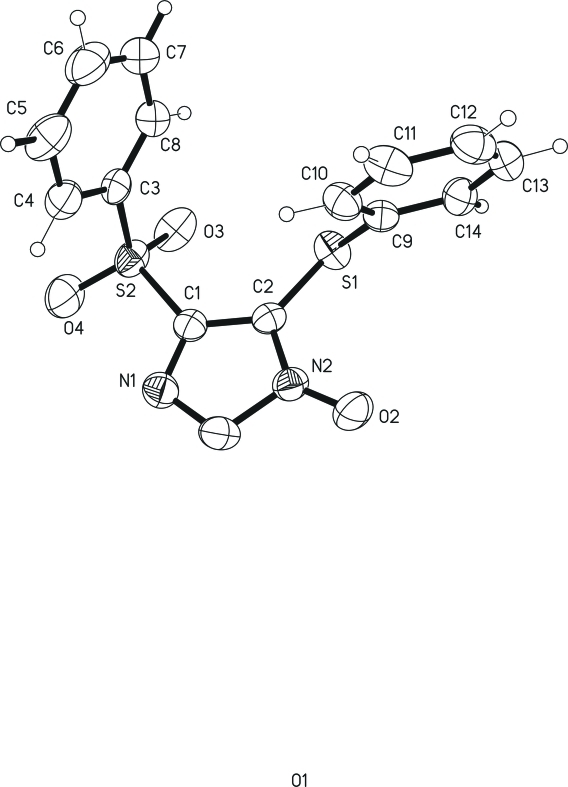
The molecular structure of the title compound showing the atomic numbering and 30% probability displacements ellipsoids.

## supplementary crystallographic information

### Comment

The title compound shows a planar moiety including the two sulfur atoms and the furoxanic ring, with a mean deviation from planarity of 0.036 Å. The planar ring contains also a significant delocalization in the N2C2C1N1O1 fragment, while the O1—N2 bond is quite greater than the corresponding N1—O1 (1.461 (3) Å *vs.* 1.363 (3) Å). The N2—O2 bond length is quite short (1.211 (3) Å), similar however to that reported by Sorba *et al.* (1996) and Dutov *et al.* (2007).

### Experimental

The 3-phenylthio-4-phenylsulfonyl-furoxanhas been obtained according to Tosco *et al.* (2004).

### Refinement

C-bound H atoms have been placed in geometrically idealized positions (C—H = 0.93 Å), and refined as riding, with *U*_iso_(H) = 1.2 *U*_eq_(C).

### Crystal data

**Table:**

C_14_H_10_N_2_O_4_S_2_	*D*_x_ = 1.497 Mg m^−^^3^
*M**_r_* = 334.36	Cu *K*α radiation, λ = 1.5418 Å
Orthorhombic, *P**n**a*2_1_	Cell parameters from 5370 reflections
*a* = 15.0182 (2) Å	θ = 3.8–62.0°
*b* = 5.5402 (1) Å	µ = 3.44 mm^−^^1^
*c* = 17.8280 (2) Å	*T* = 293 K
*V* = 1483.36 (4) Å^3^	Prismatic, colorless
*Z* = 4	0.20 × 0.16 × 0.14 mm
*F*(000) = 688	

### Data collection

**Table:**

Gemini R Ultra diffractometer	2255 independent reflections
Radiation source: Ultra (Cu) X-ray Source	2134 reflections with *I* > 2σ(*I*)
mirror	*R*_int_ = 0.023
Detector resolution: 10.2890 pixels mm^-1^	θ_max_ = 62.2°, θ_min_ = 5.0°
f scans	*h* = −17→16
Absorption correction: multi-scan (*CrysAlis RED*; Oxford Diffraction, 2008)	*k* = −6→5
*T*_min_ = 0.836, *T*_max_ = 1.000	*l* = −20→20
7933 measured reflections	

### Refinement

**Table:**

Refinement on *F*^2^	Secondary atom site location: difference Fourier map
Least-squares matrix: full	Hydrogen site location: inferred from neighbouring sites
*R*[*F*^2^ > 2σ(*F*^2^)] = 0.030	H-atom parameters constrained
*wR*(*F*^2^) = 0.084	*w* = 1/[σ^2^(*F*_o_^2^) + (0.0626*P*)^2^ + 0.0158*P*] where *P* = (*F*_o_^2^ + 2*F*_c_^2^)/3
*S* = 1.05	(Δ/σ)_max_ < 0.001
2255 reflections	Δρ_max_ = 0.20 e Å^−^^3^
199 parameters	Δρ_min_ = −0.13 e Å^−^^3^
1 restraint	Absolute structure: Flack (1983), 1039 Friedel pairs
Primary atom site location: structure-invariant direct methods	Flack parameter: 0.010 (17)

### Special details

**Table:**

Geometry. All e.s.d.'s (except the e.s.d. in the dihedral angle between two l.s. planes) are estimated using the full covariance matrix. The cell e.s.d.'s are taken into account individually in the estimation of e.s.d.'s in distances, angles and torsion angles; correlations between e.s.d.'s in cell parameters are only used when they are defined by crystal symmetry. An approximate (isotropic) treatment of cell e.s.d.'s is used for estimating e.s.d.'s involving l.s. planes.
Refinement. Refinement of *F*^2^ against ALL reflections. The weighted *R*-factor *wR* and goodness of fit *S* are based on *F*^2^, conventional *R*-factors *R* are based on *F*, with *F* set to zero for negative *F*^2^. The threshold expression of *F*^2^ > σ(*F*^2^) is used only for calculating *R*-factors(gt) *etc*. and is not relevant to the choice of reflections for refinement. *R*-factors based on *F*^2^ are statistically about twice as large as those based on *F*, and *R*- factors based on ALL data will be even larger.

### Fractional atomic coordinates and isotropic or equivalent isotropic displacement parameters (Å^2^)

**Table:**

	*x*	*y*	*z*	*U*_iso_*/*U*_eq_	
C1	0.35417 (17)	0.1230 (4)	0.94838 (14)	0.0566 (5)	
C2	0.39856 (16)	0.1654 (4)	1.01631 (14)	0.0538 (5)	
C3	0.47584 (18)	0.0455 (5)	0.83468 (14)	0.0581 (6)	
C4	0.4633 (2)	0.2458 (5)	0.78924 (16)	0.0724 (7)	
H4A	0.4065	0.3080	0.7814	0.087*	
C5	0.5353 (3)	0.3495 (7)	0.7563 (2)	0.0918 (11)	
H5A	0.5277	0.4827	0.7252	0.110*	
C6	0.6182 (3)	0.2605 (8)	0.7684 (2)	0.0959 (11)	
H6A	0.6670	0.3351	0.7461	0.115*	
C7	0.6311 (2)	0.0621 (9)	0.8129 (2)	0.0975 (12)	
H7A	0.6881	0.0014	0.8203	0.117*	
C8	0.5582 (2)	−0.0488 (6)	0.84725 (17)	0.0777 (8)	
H8A	0.5658	−0.1832	0.8778	0.093*	
C9	0.56551 (16)	0.2429 (4)	1.07525 (13)	0.0555 (6)	
C10	0.58306 (19)	0.4240 (5)	1.02395 (18)	0.0676 (7)	
H10A	0.5503	0.4356	0.9798	0.081*	
C11	0.6499 (2)	0.5868 (5)	1.0393 (2)	0.0762 (8)	
H11A	0.6613	0.7112	1.0056	0.091*	
C12	0.6999 (2)	0.5685 (5)	1.1036 (2)	0.0766 (8)	
H12A	0.7448	0.6797	1.1134	0.092*	
C13	0.6829 (2)	0.3840 (6)	1.15337 (19)	0.0788 (8)	
H13A	0.7175	0.3688	1.1964	0.095*	
C14	0.6155 (2)	0.2229 (6)	1.14018 (17)	0.0684 (7)	
H14A	0.6035	0.1010	1.1746	0.082*	
O1	0.28139 (13)	0.4102 (3)	0.99925 (13)	0.0742 (5)	
O2	0.36323 (16)	0.4572 (4)	1.10686 (15)	0.0875 (7)	
O3	0.4114 (2)	−0.3036 (4)	0.91510 (14)	0.0920 (7)	
O4	0.31016 (18)	−0.0940 (5)	0.82679 (15)	0.1039 (8)	
N1	0.28650 (15)	0.2629 (5)	0.93815 (14)	0.0701 (6)	
N2	0.35456 (14)	0.3441 (4)	1.04923 (14)	0.0635 (5)	
S1	0.48529 (5)	0.01160 (11)	1.05918 (5)	0.0699 (2)	
S2	0.38327 (5)	−0.08944 (13)	0.87725 (4)	0.0696 (2)	

### Atomic displacement parameters (Å^2^)

**Table:**

	*U*^11^	*U*^22^	*U*^33^	*U*^12^	*U*^13^	*U*^23^
C1	0.0491 (13)	0.0607 (12)	0.0600 (14)	−0.0109 (10)	0.0043 (11)	0.0001 (12)
C2	0.0525 (12)	0.0502 (11)	0.0587 (14)	−0.0059 (10)	0.0079 (10)	−0.0043 (10)
C3	0.0677 (16)	0.0610 (14)	0.0456 (13)	−0.0054 (11)	−0.0020 (11)	−0.0079 (10)
C4	0.0842 (18)	0.0719 (16)	0.0610 (15)	0.0049 (14)	0.0063 (14)	0.0019 (14)
C5	0.120 (3)	0.080 (2)	0.0751 (19)	−0.008 (2)	0.030 (2)	0.0072 (16)
C6	0.091 (2)	0.120 (3)	0.076 (2)	−0.030 (2)	0.0235 (18)	−0.008 (2)
C7	0.067 (2)	0.151 (4)	0.075 (2)	0.0048 (19)	0.0035 (16)	−0.001 (2)
C8	0.0695 (19)	0.101 (2)	0.0628 (16)	0.0053 (16)	−0.0018 (13)	0.0043 (15)
C9	0.0526 (13)	0.0510 (11)	0.0631 (15)	0.0039 (9)	0.0033 (11)	0.0003 (10)
C10	0.0629 (16)	0.0666 (14)	0.0731 (17)	0.0052 (12)	−0.0055 (13)	0.0093 (13)
C11	0.0633 (17)	0.0614 (14)	0.104 (2)	0.0004 (13)	0.0009 (17)	0.0146 (15)
C12	0.0595 (16)	0.0703 (16)	0.100 (2)	−0.0051 (13)	0.0000 (16)	−0.0116 (18)
C13	0.0634 (16)	0.104 (2)	0.0688 (17)	−0.0045 (15)	−0.0104 (14)	−0.0098 (16)
C14	0.0734 (17)	0.0754 (17)	0.0564 (14)	0.0003 (14)	−0.0004 (12)	0.0070 (13)
O1	0.0579 (10)	0.0763 (11)	0.0884 (14)	0.0081 (9)	0.0033 (9)	−0.0055 (10)
O2	0.0837 (14)	0.0954 (15)	0.0832 (14)	0.0031 (11)	0.0031 (12)	−0.0354 (13)
O3	0.136 (2)	0.0522 (10)	0.0875 (15)	−0.0183 (11)	0.0244 (13)	−0.0049 (10)
O4	0.0855 (15)	0.139 (2)	0.0869 (17)	−0.0348 (15)	−0.0055 (13)	−0.0339 (14)
N1	0.0576 (12)	0.0834 (14)	0.0693 (13)	−0.0066 (11)	−0.0004 (11)	0.0002 (12)
N2	0.0566 (12)	0.0684 (12)	0.0653 (13)	−0.0045 (10)	0.0050 (10)	−0.0112 (11)
S1	0.0718 (4)	0.0536 (3)	0.0843 (5)	−0.0042 (3)	−0.0134 (4)	0.0067 (3)
S2	0.0740 (4)	0.0725 (4)	0.0624 (4)	−0.0216 (3)	0.0049 (3)	−0.0155 (3)

### Geometric parameters (Å, °)

**Table:**

C1—N1	1.291 (4)	C9—C10	1.383 (4)
C1—C2	1.402 (4)	C9—C14	1.384 (4)
C1—S2	1.784 (3)	C9—S1	1.782 (2)
C2—N2	1.327 (3)	C10—C11	1.377 (4)
C2—S1	1.734 (3)	C10—H10A	0.9300
C3—C8	1.362 (4)	C11—C12	1.374 (5)
C3—C4	1.387 (4)	C11—H11A	0.9300
C3—S2	1.752 (3)	C12—C13	1.378 (5)
C4—C5	1.358 (4)	C12—H12A	0.9300
C4—H4A	0.9300	C13—C14	1.370 (4)
C5—C6	1.357 (6)	C13—H13A	0.9300
C5—H5A	0.9300	C14—H14A	0.9300
C6—C7	1.370 (6)	O1—N1	1.363 (3)
C6—H6A	0.9300	O1—N2	1.461 (3)
C7—C8	1.396 (5)	O2—N2	1.211 (3)
C7—H7A	0.9300	O3—S2	1.429 (3)
C8—H8A	0.9300	O4—S2	1.420 (3)
			
N1—C1—C2	113.3 (2)	C11—C10—C9	118.9 (3)
N1—C1—S2	119.2 (2)	C11—C10—H10A	120.6
C2—C1—S2	127.4 (2)	C9—C10—H10A	120.6
N2—C2—C1	105.7 (2)	C12—C11—C10	121.1 (3)
N2—C2—S1	123.1 (2)	C12—C11—H11A	119.5
C1—C2—S1	130.9 (2)	C10—C11—H11A	119.5
C8—C3—C4	121.8 (3)	C11—C12—C13	119.4 (3)
C8—C3—S2	119.1 (2)	C11—C12—H12A	120.3
C4—C3—S2	119.1 (2)	C13—C12—H12A	120.3
C5—C4—C3	118.9 (3)	C14—C13—C12	120.6 (3)
C5—C4—H4A	120.6	C14—C13—H13A	119.7
C3—C4—H4A	120.6	C12—C13—H13A	119.7
C6—C5—C4	120.6 (4)	C13—C14—C9	119.5 (3)
C6—C5—H5A	119.7	C13—C14—H14A	120.3
C4—C5—H5A	119.7	C9—C14—H14A	120.3
C5—C6—C7	120.9 (3)	N1—O1—N2	107.14 (18)
C5—C6—H6A	119.6	C1—N1—O1	106.9 (2)
C7—C6—H6A	119.6	O2—N2—C2	135.1 (2)
C6—C7—C8	119.8 (4)	O2—N2—O1	117.9 (2)
C6—C7—H7A	120.1	C2—N2—O1	107.0 (2)
C8—C7—H7A	120.1	C2—S1—C9	103.02 (11)
C3—C8—C7	118.1 (3)	O4—S2—O3	120.89 (17)
C3—C8—H8A	120.9	O4—S2—C3	110.29 (15)
C7—C8—H8A	120.9	O3—S2—C3	108.92 (15)
C10—C9—C14	120.5 (2)	O4—S2—C1	105.81 (14)
C10—C9—S1	123.0 (2)	O3—S2—C1	106.53 (13)
C14—C9—S1	116.3 (2)	C3—S2—C1	102.76 (12)
